# National Surveillance-Based Retrospective Ecological Longitudinal Analysis of Stroke Incidence Trends and Health-Screening Indicators in Korea, 2011–2023, with Model-Based Projections to 2028 Using National Health Insurance Service Data

**DOI:** 10.3390/healthcare14131815

**Published:** 2026-06-23

**Authors:** Hyeran Jung, Minsun Jung

**Affiliations:** Department of Pathology, Yonsei University College of Medicine, Seoul 03722, Republic of Korea

**Keywords:** stroke, incidence, National Health Insurance Service, health screening, Korea, longitudinal trend, prediction model, public health policy

## Abstract

**Background**: Stroke remains a leading cause of mortality, disability, and health-system burden in Korea’s rapidly aging population. We aimed to describe national stroke incidence trends from 2011 to 2023, characterize ecological associations between stroke incidence and health-screening indicators, and generate model-based projections through 2028 to support health-system planning. **Methods**: This retrospective ecological longitudinal analysis used three publicly available aggregate national data sources: (1) NHIS annual aggregate statistics on crude and age-standardized stroke incidence, stroke case counts, first-onset vs. recurrent stroke, and case-fatality rates (2011–2023); (2) regional standardized health-awareness survey rates for stroke symptoms, myocardial infarction symptoms, blood pressure, and blood glucose (2017–2025); and (3) national cancer-screening outcome tallies for breast and cervical cancer (2010–2024). All analyses used pre-aggregated annual summary data; individual-level NHIS records were not used. Annual trends were modeled with ordinary least-squares linear regression (n = 13 annual observations). Pearson correlations were computed only for overlapping observation windows. Model-based projections are presented with 95% prediction intervals and are explicitly distinguished from observed NHIS values. This study is purely descriptive and ecological; no causal inference is made. **Results**: Crude stroke incidence increased from 199.2 to 221.1 per 100,000 (2011–2023; slope +2.32/year, R^2^ = 0.83), whereas age-standardized incidence declined from 158.3 to 113.2 per 100,000 (slope −3.41/year, R^2^ = 0.96), a pattern consistent with demographic aging as a contributing factor to growing absolute burden, though formal age-decomposition analysis would be required to confirm this inference. Total cases increased from 99,837 to 113,098; the 30-day case-fatality rate declined from 8.5% to 7.5%. Ecological correlations showed that blood glucose awareness was strongly negatively correlated with age-standardized incidence (r = −0.944, *p* = 0.001, n = 7), though these are ecological associations and must not be interpreted as individual-level causal relationships. Model-based projections estimate crude incidence near 230.7 (95%PI 219.2–242.2) and age-standardized incidence near 103.2 (95%PI 95.7–110.8) per 100,000 by 2026. **Conclusions**: Concurrent increase in crude burden and decline in age-standardized incidence reflects demographic aging as the primary driver of Korea’s stroke burden. Projections support integrated cardiovascular prevention, public health education, and age-sensitive service planning. All projections are short-horizon statistical extrapolations intended for policy scenario planning only and must not be interpreted as observed future NHIS outcomes.

## 1. Introduction

Stroke remains one of the leading causes of mortality, disability, and health-system burden globally and in Korea [1,2]. According to the GBD 2021 Stroke Risk Factor Collaborators, stroke accounted for approximately 7.3 million deaths worldwide in 2021, with the highest incidence-rate increases in East Asia [1]. In Korea, which formally entered super-aged society status in 2025, the absolute number of stroke patients has grown substantially owing to rapid demographic aging even as age-standardized incidence rates have declined, reflecting improvements in primary prevention and acute care. This divergence between crude and age-standardized incidence is epidemiologically important: crude incidence reflects the actual patient volume that the healthcare system must manage, whereas age-standardized incidence isolates biological or behavioral risk independent of demographic shifts. Characterizing both metrics together with national health-screening indicators is therefore essential for comprehensive health-system planning. Prior Korean studies have examined stroke incidence and risk factors using NHIS-linked administrative data, but most focused on specific cohort subsets or earlier calendar periods and did not integrate multi-domain health-screening indicators simultaneously with projections [3,4]. National aggregate data on health awareness (stroke symptoms, blood pressure, blood glucose) and cancer-screening outcomes are available through the Korean Community Health Survey and NHIS screening reports and may provide useful ecological context for surveillance, even if they cannot be linked to individual stroke events without individual-level data.

Female-specific cancer-screening indicators (breast and cervical cancer) were included as contextual screening variables because prior research has identified plausible intersections between cancer survivorship and stroke risk. Specifically, radiation-induced vascular injury, cardiotoxic chemotherapy, thrombogenic effects of hormone therapy, and coagulation changes during the menopausal transition have all been associated with increased cerebrovascular risk in cancer survivors [5,6,7,8]. However, the present study uses only aggregate national screening outcome tallies (not confirmed cancer diagnoses or treatment records) and cannot directly assess these mechanisms at the individual level. These variables are therefore retained as surveillance context indicators only, not as causal predictors. In aggregate ecological analysis, a correlation between screening indicators and stroke incidence should be interpreted as an association across time periods at the population level, subject to ecological fallacy, and not as evidence of an individual-level causal pathway.

This study had four objectives: (1) to describe national 2011–2023 crude and age-standardized stroke incidence trends, case counts, first-onset vs. recurrent incidence, and case-fatality rates using NHIS aggregate statistics; (2) to examine ecological associations between stroke incidence and available health-screening and awareness indicators over overlapping observation periods; (3) to generate model-based projections of stroke burden through 2028 with uncertainty intervals for health-system planning; and (4) to assess these findings in the context of their ecological design limitations in accordance with STROBE reporting guidelines for observational studies [9]. This study was additionally conducted and reported in accordance with the Strengthening the Reporting of Observational Studies in Epidemiology (STROBE) guidelines for ecological observational studies; data provenance, variable definitions, and observed-vs-projected separation follow STROBE principles throughout.

## 2. Materials and Methods

### 2.1. Study Design and Data Sources

This was a national surveillance-based retrospective ecological longitudinal analysis using three categories of publicly available aggregate Korean national health-statistics datasets. This study was conducted and reported in accordance with the Strengthening the Reporting of Observational Studies in Epidemiology (STROBE) guidelines for ecological observational studies [9]. Data source 1: NHIS annual aggregate stroke statistics (2011–2023). The primary data source was the NHIS annual aggregate statistical reports, specifically the national stroke incidence and case-fatality datasets publicly accessible via the Korean Statistical Information Service (KOSIS; https://kosis.kr) [3]. These files provide pre-aggregated annual totals by sex, age group, and stroke type (first-onset vs. recurrent) for crude incidence rate (per 100,000 population), age-standardized incidence rate (per 100,000), total stroke case counts, 30-day case-fatality rate (%), and 1-year case-fatality rate (%). The NHIS stroke case definition uses ICD-10 codes I60–I64 applied to insurance claims. Age standardization was performed by NHIS using the 2005 Korean census standard population. These are administrative surveillance statistics derived from insurance claims, not clinically adjudicated diagnoses; accordingly, some degree of misclassification is possible. Data source 2: Regional health-awareness survey aggregates (2017–2025). Standardized awareness rates for stroke early-symptom recognition, myocardial infarction early-symptom recognition, blood pressure value awareness, and blood glucose value awareness were obtained from the Korean Community Health Survey aggregate tables published by the Korea Disease Control and Prevention Agency (KDCA), available via KOSIS [3]. These files provide annual municipal-level standardized rates (%) with standard errors. For this ecological analysis, national-level aggregates were used as the primary unit of analysis. Data source 3: National cancer-screening outcome tallies (2010–2024). Annual national aggregate counts of breast cancer and cervical cancer screening result categories (no abnormality, benign lesion, suspected malignancy, etc.) were obtained from the NHIS national cancer-screening statistics published via KOSIS [4]. Suspected malignancy counts were used as contextual screening utilization indicators; these represent administrative screening outcomes and are not equivalent to confirmed cancer diagnoses or individual-level clinical outcomes. Observed stroke incidence values are available for 2011–2023 only; values for 2024–2028 are model-based projections and must not be interpreted as observed NHIS incidence data.

### 2.2. Variables

The primary outcomes were crude stroke incidence per 100,000 population, age-standardized stroke incidence per 100,000 population, total stroke cases, and case-fatality rates. Secondary indicators included sex-specific incidence, age-specific incidence, weighted provincial awareness rates, and suspected cancer-screening counts among adults aged 55 years or older. Screening variables were interpreted only as screening indicators and not as confirmed disease prevalence.

### 2.3. Statistical Analysis

Annual descriptive statistics were calculated for all observed variables. This study is purely descriptive and ecological; no inferential causal claims are made, and all statistical tests should be interpreted as exploratory. Trend modeling and projections: Linear ordinary least-squares (OLS) time-trend models were fitted separately for each outcome (crude incidence, age-standardized incidence, total cases, 30-day case-fatality rate, 1-year case-fatality rate) using calendar year as the independent variable (n = 13 annual observations, 2011–2023). The linearity assumption was justified by visual inspection of observed trends and residual plots; no systematic curvature was detected in the series. Model diagnostics included coefficient of determination (R^2^), Akaike information criterion (AIC), annual slope with standard error, t-statistic, and *p*-value. Residual homoscedasticity and absence of strong autocorrelation were confirmed visually; formal Durbin–Watson testing was not performed given the short series length. Model-based projections were generated for 2024–2028 with 95% observation-level prediction intervals derived from the OLS forecast variance. The linearity assumption may not hold beyond the observed range; these projections should be treated as short-horizon descriptive extrapolations for policy scenario planning, not as definitive predictions. As more annual data points become available, application of ARIMA or damped-trend exponential smoothing models would be appropriate to capture potential non-linear trends. Ecological correlation analysis: Pearson correlation coefficients were calculated between age-standardized stroke incidence and each health-awareness or cancer-screening indicator, restricted to overlapping observed years (awareness indicators: 2017–2023, n = 7; cancer-screening indicators: 2011–2023, n = 13). These ecological correlations are exploratory and subject to ecological fallacy: a correlation between aggregate population-level trends does not establish individual-level causal associations and may reflect shared secular trends, confounding by demographic or socioeconomic changes, or other third-variable effects. Correlation findings are therefore presented in supplementary context rather than as primary results. These aggregate time-series correlations cannot support individual-level causal inference and must not be interpreted as evidence of causal pathways at the individual patient level. To summarize the analytical workflow: (1) observed annual data were compiled by domain and year; (2) OLS linear models were fitted separately for each primary outcome using calendar year as the sole predictor; (3) residual plots and R^2^ were inspected to assess linearity; (4) Pearson correlations were computed for each indicator pair restricted to overlapping years; and (5) 95% prediction intervals were generated from OLS forecast variance for all projected years. Full model diagnostic outputs (R^2^, AIC, slope, SE, t-statistic, *p*-value, and residual inspection results) and ecological correlation tables are provided in the Supplementary Materials (Tables S1 and S2), and readers are directed there for complete methodological transparency. Statistical analyses were performed using Python 3.11 with pandas, scipy, and statsmodels libraries. All statistical tests were two-sided; *p*  <  0.05 was considered nominally significant, though given the small sample sizes and ecological design, all *p*-values should be interpreted with caution [9,10,11,12].

### 2.4. Ethical Considerations

Ethical review and approval were waived by the Institutional Review Board of Chungnam National University (IRB No. 202506-SB-105-01) as the study used deidentified secondary data.

## 3. Results

### 3.1. Data Availability and Variable Structure

The available data domains, observation periods, and analytic uses are summarized in Table 1.

**Table 2:** Model-based projections of stroke burden, 2024–2028 (not observed NHIS values; OLS linear extrapolation with 95% prediction intervals; intended for policy scenario planning only).

Outcome	Year	Predicted	Lower 95	Upper 95
Crude stroke incidence	2026	230.7	219.2	242.2
Crude stroke incidence	2027	233.0	221.1	244.9
Crude stroke incidence	2028	235.3	223.0	247.7
Age-standardized stroke incidence	2026	103.2	95.7	110.8
Age-standardized stroke incidence	2027	99.8	92.0	107.6
Age-standardized stroke incidence	2028	96.4	88.3	104.5
Total stroke cases	2026	119,354.1	113,052.6	125,655.7
Total stroke cases	2027	120,723.2	114,209.1	127,237.3
Total stroke cases	2028	122,092.4	115,351.1	128,833.6
30-day case fatality	2026	6.910	5.950	7.870
30-day case fatality	2027	6.840	5.840	7.830
30-day case fatality	2028	6.760	5.730	7.790
1-year case fatality	2026	18.7	16.1	21.3
1-year case fatality	2027	18.7	16.0	21.4
1-year case fatality	2028	18.7	15.9	21.5

Note: Prediction intervals are 95% observation-level intervals from ordinary least-squares trend models.

### 3.2. Observed Stroke Incidence and Fatality Trends

**Figure 1:**
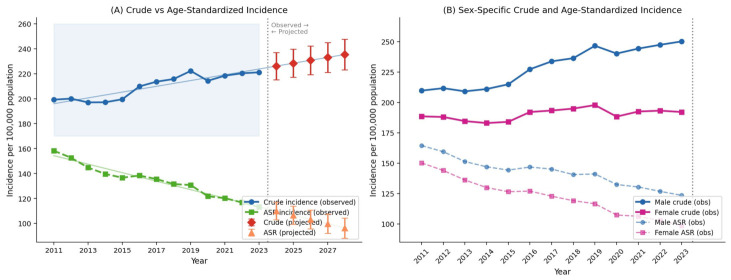
National stroke incidence trends and model-based projections, South Korea, 2011–2023 (observed) and 2024–2028 (model-based projections). Panel (**A**): Crude incidence rate (per 100,000) and age-standardized incidence rate (per 100,000) with 95% prediction intervals on projected values; vertical dashed line separates observed from projected data. Panel (**B**): Sex-specific crude and age-standardized incidence rates, 2011–2023. Solid symbols = observed; open symbols with error bars = model-based projections.

**Figure 2:**
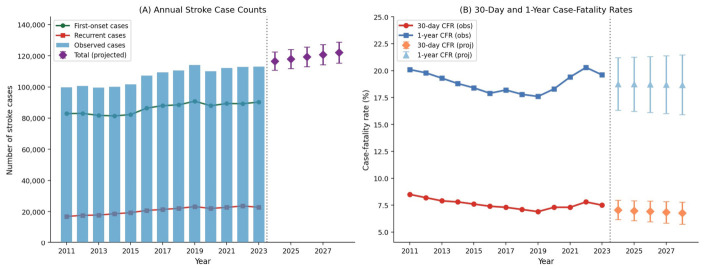
Annual stroke case counts and case-fatality rates, South Korea, 2011–2023 (observed) and 2024–2028 (model-based projections). (**A**) Total annual stroke case counts (bars, observed), first-onset cases, and recurrent cases with model-based projected total (diamonds, 95% PI). (**B**) 30-day and 1-year case-fatality rates (%) with model-based projections and 95% prediction intervals. Vertical dashed line separates observed from projected data in both panels.

Crude stroke incidence increased from 199.2 per 100,000 in 2011 to 221.1 per 100,000 in 2023. In contrast, age-standardized incidence declined from 158.3 to 113.2 per 100,000. Total stroke cases increased from 99,837 to 113,098, indicating that demographic aging and absolute case burden remain important even as age-standardized risk declines (Table 3; Figure 1 and Figure 2).

### 3.3. Trend Models and Predictions to 2028

Linear time-trend model estimates and model-based predictions are presented in Table 2 and Table 4.

The age-standardized incidence model showed a negative annual slope of −3.41 per 100,000 per year, while the crude incidence model showed a positive annual slope of 2.32 per 100,000 per year. This divergence supports the interpretation that population aging is contributing substantially to the national stroke burden (Figure 1).

### 3.4. Age-Specific and Health-Screening Indicators

**Figure 3:**
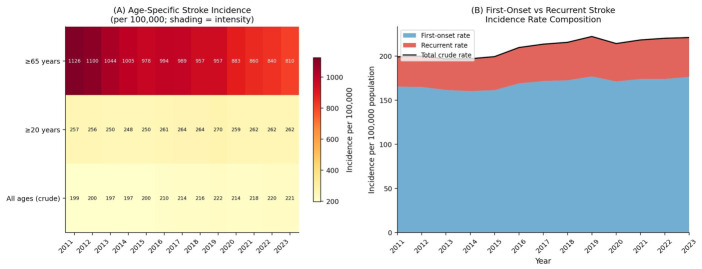
Age-specific stroke incidence rates and first-onset vs. recurrent stroke trends, South Korea, 2011–2023. (**A**) Heatmap of age-specific crude incidence rates (per 100,000) for the ≥65-year, ≥20-year, and all-ages (crude) strata across observation years; shading intensity reflects incidence magnitude. (**B**) Stacked area chart showing the annual composition of first-onset and recurrent stroke incidence rates; the growing recurrent stroke proportion highlights the urgency of secondary prevention.

**Figure 4:**
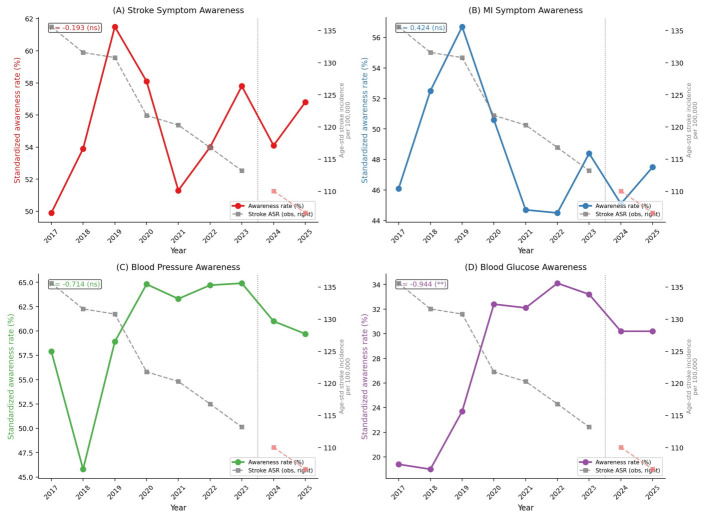
Health-awareness indicators with age-standardized stroke incidence overlay, South Korea, 2017–2025. (**A**–**D**) show annual standardized rates (%) for stroke symptom awareness, myocardial infarction symptom awareness, blood pressure value awareness, and blood glucose value awareness, respectively, overlaid with observed age-standardized stroke incidence (gray dashed line, right axis). Pearson correlation coefficient (r) with significance label shown in each panel (n = 7 overlapping years, 2017–2023). Values for 2024–2025 stroke ASR are model-based projections (light coral markers). All correlations are ecological aggregate-level associations and must not be interpreted as evidence of individual-level causal relationships. ** *p* < 0.001; ns, not significant (*p* ≥ 0.05).

**Figure 5:**
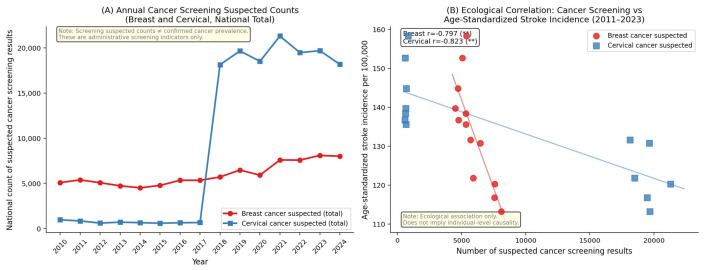
National cancer-screening indicators and ecological correlation with age-standardized stroke incidence, South Korea, 2010–2024. Panel (**A**): Annual national aggregate counts of breast cancer and cervical cancer suspected screening results (2010–2024); these are administrative screening outcome tallies, not confirmed cancer diagnoses. Panel (**B**): Scatter plot showing the ecological correlation between suspected cancer-screening counts and age-standardized stroke incidence (2011–2023); regression lines overlaid with Pearson r values (breast r = −0.797, *p* = 0.001; cervical r = −0.823, *p* = 0.001). Note: These are aggregate ecological associations susceptible to ecological fallacy and shared secular trends; they must not be interpreted as evidence of individual-level causal relationships between cancer screening and stroke risk. ** *p* < 0.001.

Age-specific stroke incidence patterns, health-awareness indicators, ecological correlations (exploratory only; n = 7 for awareness indicators), and national cancer-screening indicators are shown in Figure 3, Figure 4 and Figure 5 and Table 5. All correlation findings are presented as supplementary ecological context and must not be interpreted as evidence of individual-level causal relationships.

## 4. Discussion

This retrospective ecological longitudinal analysis of Korean national stroke statistics documents four principal findings. First, crude stroke incidence and total case counts increased substantially between 2011 and 2023; this pattern is consistent with an ecological signal of demographic aging, as suggested by the concurrent decline in age-standardized incidence, though formal age decomposition was not performed and this interpretation remains an inference. Second, sex-stratified analysis shows consistently higher crude incidence in males, though female incidence remains substantial and both sexes contributed to the growing absolute burden. Third, the first-onset to recurrent stroke ratio increased over time, with recurrent strokes accounting for approximately 20% of annual cases by 2023, underscoring the importance of secondary prevention. Fourth, model-based projections indicate that crude incidence and absolute case counts are likely to continue increasing through 2028 under current trends, while age-standardized incidence may continue declining.

The divergence between crude and age-standardized stroke incidence is consistent with international trends documented in the GBD 2021 Stroke Risk Factor Collaborators report, which found that absolute stroke cases continued to increase in East Asia despite declining age-standardized rates [1]. Similar patterns have been documented in Japan, China, and Taiwan, where rapid demographic aging accelerated absolute burden even as public health and treatment improvements suppressed age-specific risk. The claim that population aging is a plausible contributor is based on this epidemiological pattern of diverging crude and age-standardized trends; however, the present analysis does not perform a formal age decomposition and this should be considered an inference supported by the pattern of results, not a demonstrated quantitative finding. Full OLS model diagnostic outputs (R^2^, AIC, slope SE, residual plots) supporting this and all other trend findings are reported in Supplementary Table S1. Future work with age-decomposition methods would be needed to formally partition the contributions of aging and risk-factor change to trend changes.

The ecological correlation analysis showed that blood glucose awareness was strongly negatively correlated with age-standardized stroke incidence over 2017–2023 (r = −0.944, *p* = 0.001), and blood pressure awareness showed a moderate negative correlation (r = −0.714, *p* = 0.072). These associations are plausible from a public health perspective—improved population awareness of blood glucose and blood pressure values is a precursor to hypertension and diabetes control, both of which are major modifiable risk factors for stroke [2]. However, given the ecological design and small overlapping sample size (n = 7 years), these findings are exploratory associations at the aggregate level and must not be interpreted as evidence of individual-level causal relationships. The observed correlations are susceptible to ecological fallacy, shared secular trends, and unmeasured confounders. Complete ecological correlation coefficients, confidence intervals, and overlapping observation windows for all awareness and cancer-screening indicators are provided in Supplementary Table S2; readers are directed there for the full evidence base underlying the correlation findings reported here. Similarly, the breast cancer suspected screening count showed a negative ecological correlation with age-standardized stroke incidence (r = −0.797, *p* = 0.001) and cervical cancer suspected count showed a negative correlation (r = −0.823, *p* = 0.001) over 2011–2023 (Supplementary Table S2). These associations likely reflect shared temporal trends (both improving over time) rather than a biological or mechanistic relationship; they should be used for hypothesis generation in future individual-level studies only. Cancer survivorship and cerebrovascular risk intersections—through radiation-induced vascular injury, cardiotoxic chemotherapy, hormonal therapy, and menopausal transition—remain biologically plausible but cannot be evaluated from these aggregate data [5,6,7,8].

Model-based projections through 2028 suggest continued pressure on the Korean stroke care system: crude incidence is projected to reach approximately 230.7 (95%PI 219.2–242.2) per 100,000 by 2026, and total cases may approach 119,354 (95%PI 113,053–125,656). Full projected values for 2024–2028 with 95% prediction intervals for all outcomes are listed in Table 2; the underlying OLS model diagnostics are detailed in Supplementary Table S1. These projections should be interpreted as short-horizon planning estimates derived from linear extrapolation of 13 observed data points. The OLS model assumes that the observed linear trend will continue, which may not hold if structural changes occur in demographic aging speed, treatment access, or prevention programs. Once 2024 and 2025 observed NHIS data are released, model-based forecasts should be validated against these values to confirm prediction interval coverage and model adequacy. The availability of additional data points would also support the application of ARIMA or exponential smoothing forecasting methods, which may better capture non-linear trend dynamics [13].

From a policy perspective, these findings support a dual strategy. First, continued prevention targeting modifiable cardiovascular risk factors—hypertension, diabetes, dyslipidemia, smoking, and physical inactivity—is essential to sustain the declining age-standardized trend [2]. The Lancet Commission on dementia prevention, intervention, and care identified 12 modifiable risk factors responsible for approximately 40% of preventable dementia and stroke burden, many of which are addressable through national health-screening programs. Second, health-system planning must anticipate growing absolute demand for emergency stroke care, rehabilitation, long-term care, and secondary prevention services as the older adult population expands. The increase in recurrent stroke cases over the observation period specifically highlights the urgency of post-stroke secondary prevention including antiplatelet or anticoagulant therapy, blood pressure management, and lifestyle modification. Policy recommendations in this ecological study should be framed cautiously and remain proportional to the descriptive nature of the evidence; causal attribution of trends to specific interventions requires individual-level data [12].

## 5. Limitations

This study has several important limitations that must be considered when interpreting the findings. First, and most fundamentally, this analysis used pre-aggregated annual national statistics and did not use individual-level NHIS raw data files. Therefore, the analysis cannot control for any individual-level confounding factors including medication exposure (antihypertensives, anticoagulants, antiplatelet agents), treatment modality, smoking history, alcohol consumption, obesity, socioeconomic status, or individual cancer survivorship status. This is the critical constraint of the ecological design: results describe population-level associations across time and cannot be extrapolated to individual risk. Second, the study is susceptible to ecological fallacy. The finding that aggregate stroke incidence correlates ecologically with aggregate health-awareness rates does not mean that individuals with higher health awareness have lower individual stroke risk, nor does it exclude confounding by shared secular trends (e.g., improvements in healthcare access, dietary change, aging demographics) that affect both variables simultaneously. These correlations are exploratory surveillance indicators only. Third, observed stroke incidence data were available only through 2023; values for 2024–2028 are model-based projections and must NOT be described as observed NHIS outcomes. All values in this table are model-generated extrapolations intended for policy scenario planning only. The observed versus projected distinction is clearly labeled in all tables and figures throughout this manuscript. Fourth, cancer-screening suspected counts are administrative screening outcomes (not confirmed cancer diagnoses or treatment records) and are not equivalent to individual cancer prevalence or survivorship status. The inclusion of these variables as contextual ecological indicators is justified by the theoretical plausibility of cancer–cardiovascular intersections [5,6,7,8], but these cannot be tested as causal predictors in this dataset. Fifth, linear OLS trend models were applied to 13 annual observations. This short series limits statistical power for detecting non-linear trends and precludes the use of more complex time-series models. The linearity assumption may not hold if demographic, treatment, or behavioral changes produce structural breaks in the trend. The projections should therefore be treated as short-horizon descriptive extrapolations intended for policy scenario planning, not as definitive predictions. Validation of the 2024 and 2025 projections against official NHIS data when available is strongly recommended. Sixth, the claim that population aging is a major driver of the increase in absolute stroke cases is based on the observed pattern of diverging crude and age-standardized incidence, not on a formal age-decomposition analysis. This remains an inference rather than a demonstrated quantitative finding. Seventh, the NHIS stroke case definition uses administrative ICD-10 coding from insurance claims, which may be subject to misclassification, coding changes over time, and differences in healthcare utilization patterns. Eighth, this study does not account for potential changes in stroke treatment during the observation period (e.g., expansion of thrombectomy centers, changes in thrombolysis rates) that could influence case-fatality trends independently of incidence changes.

## 6. Conclusions

Using publicly available Korean national health statistics, this ecological longitudinal analysis demonstrates that crude stroke incidence and total case burden increased substantially from 2011 to 2023, while age-standardized incidence declined concurrently. This divergence is consistent with a role of demographic aging in growing absolute stroke burden; this interpretation is inferential in the absence of formal age decomposition, and formal age-decomposition analysis is needed to quantify this contribution. Ecological correlations between health-awareness indicators and age-standardized stroke incidence are exploratory associations at the aggregate level and must not be interpreted as individual-level causal relationships. Model-based projections through 2028 suggest that absolute stroke case burden may continue to grow, supporting a dual policy strategy of sustained cardiovascular prevention targeting modifiable risk factors and proactive health-system capacity planning for emergency stroke care, rehabilitation, and secondary prevention. All projections are short-horizon statistical extrapolations; each is intended for policy scenario planning only and must not be interpreted as a definitive prediction of future NHIS-observed incidence; validation against official NHIS data for 2024–2025 is recommended once available. Future research using individually approved NHIS cohort data with individual-level confounding adjustment is required to validate causal pathways, assess the role of cancer survivorship and treatment in stroke risk, and develop clinically applicable individual risk prediction models.

## Figures and Tables

**Table 1:** Data availability and analytic variables.

Data Domain	Available Years in Attached Files	Analytic Use
Stroke incidence and case counts	2011–2023	Crude, age-standardized, sex-specific, and age-specific rates
Stroke case fatality	2011–2023	30-day and 1-year case-fatality rates
Health-awareness indicators	2017–2025	Weighted provincial standardized rates
Cancer-screening indicators	2010–2024	Suspected breast/cervical cancer screening counts among age ≥ 55
Forecast outcomes	2024–2028	Model-based predictions; 2026–2028 emphasized

Note: Observed stroke incidence data are available for 2011–2023 only. Values for 2024–2028 in Table 2 are model-based projections derived from OLS linear extrapolation and must not be interpreted as observed NHIS incidence data. This table serves as the authoritative master timeline reference for the entire manuscript.

**Table 3:** Observed annual stroke incidence, cases, and case-fatality measures, South Korea, 2011–2023 (all values are observed NHIS statistics; no model-based projections are included in this table).

Year	Stroke Cases	Crude Incidence per 100,000	Age-Standardized Incidence per 100,000	Male Crude Incidence	Female Crude Incidence	30-Day Fatality (%)	1-Year Fatality (%)
2011	99,837	199.2	158.3	209.8	188.6	8.500	20.1
2012	100,688	200.0	152.7	211.8	188.1	8.200	19.8
2013	99,596	197.0	144.8	209.2	184.7	7.900	19.3
2014	100,056	197.1	139.7	211.1	183.1	7.800	18.8
2015	101,648	199.5	136.7	215.0	184.1	7.600	18.4
2016	107,210	209.8	138.4	227.4	192.2	7.400	17.9
2017	109,445	213.6	135.6	233.9	193.4	7.300	18.2
2018	110,652	215.7	131.6	236.5	195.0	7.100	17.8
2019	114,097	222.2	130.8	246.7	197.9	6.900	17.6
2020	110,031	214.3	121.8	240.3	188.4	7.300	18.3
2021	112,123	218.4	120.3	244.4	192.6	7.300	19.4
2022	112,936	220.3	116.8	247.5	193.3	7.800	20.3
2023	113,098	221.1	113.2	250.3	192.2	7.500	19.6

Note: Rates are per 100,000 population unless otherwise specified. Fatality values are percentages.

**Table 4:** Linear time-trend models for observed outcomes.

Outcome	n	Annual Slope	SE	t	*p*-Value	R^2^	AIC
Crude stroke incidence	13	2.315	0.315	7.343	0.000	0.831	76.4
Age-standardized stroke incidence	13	−3.409	0.205	−16.6	0.000	0.962	65.2
Total stroke cases	13	1369.1	172.0	7.959	0.000	0.852	240.2
30-day case fatality	13	−0.075	0.026	−2.845	0.016	0.424	11.7
1-year case fatality	13	−0.018	0.071	−0.256	0.803	0.006	37.6

Note: Slope indicates annual change in the outcome. Forecasts should be interpreted as short-horizon statistical projections rather than confirmed future observations.

**Table 5:** Pearson correlations between age-standardized stroke incidence and available screening/awareness indicators.

Indicator	From	To	n	Pearson r	*p*-Value
Stroke symptom awareness	2017	2023	7	−0.193	0.679 (ns)
MI symptom awareness	2017	2023	7	0.424	0.343 (ns)
Blood pressure awareness	2017	2023	7	−0.714	0.072 (ns)
Blood glucose awareness	2017	2023	7	−0.944	<0.001 **
Breast cancer-suspected screening count ≥55	2011	2023	13	−0.797	<0.001 **
Cervical cancer-suspected screening count ≥55	2011	2023	13	−0.823	0.001 **

Note: Correlations were calculated only for overlapping observed calendar years. Awareness indicators: 2017–2023 (n = 7); cancer-screening indicators: 2011–2023 (n = 13). These are ecological associations at the aggregate population level and are susceptible to ecological fallacy, shared secular trends, and unmeasured confounding. They must not be interpreted as evidence of individual-level causal relationships. ** *p* < 0.01; ns = not significant at α = 0.05. All *p*-values should be interpreted with caution given small sample sizes.

## Data Availability

The analysis was based on the attached aggregate raw data files provided for manuscript preparation. Individual-level NHIS prevalence raw data were not used in this study. All analyses were performed exclusively on pre-aggregated annual national statistics. Individual-level NHIS data access requires separate institutional approval from the NHIS Research Institute (https://www.nhis.or.kr/); such data were not accessed or analyzed for this manuscript. Aggregate national data used in this analysis are publicly available via KOSIS (https://kosis.kr) and NHIS (https://www.nhis.or.kr/) and may be accessed by researchers upon reasonable request subject to data-use terms. Aggregate national data may be available from relevant Korean public health data portals or from the authors upon reasonable request, subject to data-use permissions.

## Supplementary Materials

The following supporting information can be downloaded at: https://www.mdpi.com/article/10.3390/healthcare14131815/s1, Table S1: Annual observed stroke incidence, case counts, and case-fatality rates by sex, South Korea, 2011–2023; Table S2: Ecological correlation variables—annual health-awareness and cancer-screening indicator data, South Korea, 2010–2025.
