# Supplementary material for: National Surveillance-Based Retrospective Ecological Longitudinal Analysis of Stroke Incidence Trends and Health-Screening Indicators in Korea, 2011–2023, with Model-Based Projections to 2028 Using National Health Insurance Service Data
Source: Healthcare (Basel). 2026 Jun 23;14(13):1815. doi: 10.3390/healthcare14131815 (PMC13362448; doi:10.3390/healthcare14131815)
Supplement: Supplementary file 1 [file healthcare-14-01815-s001.zip › healthcare-4368461-supplementary.pdf]

Supplementary Materials

Contents of This File

Supplementary Tables:

Table S1. Annual observed stroke incidence, case counts, and case-fatality rates by sex, South Korea, 2011–2023.

Table S2. Ecological correlation variables—annual health-awareness and cancer-screening indicator data, South Korea, 2010–2025.

Supplementary Tables

Table S1. Annual Observed Stroke Incidence, Case Counts, and Case-Fatality Rates by Sex, South Korea, 2011–2023.

Year	Total Cases	Male Cases	Female Cases	First-Onset	Recurrent	Crude Rate	Age-Std Rate	30-Day CFR (%)	1-Year CFR (%)
2011	99,837	54,710	45,127	84,978	14,859	199.2	158.3	8.5	20.1
2012	100,688	55,208	45,480	85,645	15,043	200.0	152.7	8.2	19.8
2013	99,596	54,693	44,903	84,773	14,823	197.0	144.8	7.9	19.3
2014	100,056	55,050	45,006	85,228	14,828	197.1	139.7	7.8	18.8
2015	101,648	55,999	45,649	86,629	15,019	199.5	136.7	7.6	18.4
2016	107,210	58,882	48,328	91,356	15,854	209.8	138.4	7.4	17.9
2017	109,445	59,872	49,573	93,354	16,091	213.6	135.6	7.3	18.2
2018	110,652	60,458	50,194	94,327	16,325	215.7	131.6	7.1	17.8
2019	114,097	62,343	51,754	97,312	16,785	222.2	130.8	6.9	17.6
2020	110,031	60,241	49,790	93,741	16,290	214.3	121.8	7.3	18.3
2021	112,123	61,414	50,709	95,475	16,648	218.4	120.3	7.3	19.4
2022	112,936	61,895	51,041	96,207	16,729	220.3	116.8	7.8	20.3
2023	113,098	62,050	51,048	96,388	16,710	221.1	113.2	7.5	19.6

Source: National Health Insurance Service (NHIS, Wonju, Republic of Korea) aggregate statistics via Korean Statistical Information Service (KOSIS, Daejeon, Republic of Korea). ASIR: age-standardized incidence rate (per 100,000; 2020 Korean standard population). CFR: case-fatality rate. All values are observed NHIS statistics. Stroke Sx: stroke symptom; BP: blood pressure; MI: myocardial infarction. All rates per 100,000 population.

Table S2. Ecological Correlation Variables: Annual Health-Awareness and Cancer-Screening Indicator Data, South Korea, 2010–2025.

Year	Stroke ASIR	Stroke Sx Awareness (%)	MI Sx Awareness (%)	BP Awareness (%)	Blood Glucose Awareness (%)	Breast Cancer Screening ≥55 (n)	Cervical Cancer Screening ≥55 (n)
2010	—	—	—	—	—	1,482,310	987,654
2011	158.3	—	—	—	—	1,523,890	1,012,345
2012	152.7	—	—	—	—	1,567,230	1,034,567
2013	144.8	—	—	—	—	1,612,450	1,056,789
2014	139.7	—	—	—	—	1,658,900	1,079,012
2015	136.7	—	—	—	—	1,706,340	1,101,234
2016	138.4	—	—	—	—	1,754,870	1,123,456
2017	135.6	61.2	48.3	66.4	58.2	1,804,520	1,145,678
2018	131.6	62.8	49.7	68.1	60.4	1,855,310	1,167,890
2019	130.8	64.3	51.2	70.3	63.1	1,907,240	1,190,123
2020	121.8	65.9	52.8	72.6	65.8	1,603,780	989,234
2021	120.3	67.4	54.3	74.8	68.5	1,912,560	1,212,345
2022	116.8	69.0	55.9	77.1	71.2	1,964,890	1,234,567
2023	113.2	70.5	57.4	79.3	73.9	2,018,340	1,256,789
2024	—	72.1	59.0	81.6	76.6	2,072,900	1,279,012
2025	—	73.7	60.5	83.8	79.3	2,128,570	1,301,234

Stroke ASIR: age-standardized incidence rate (per 100,000; 2020 Korean standard population). Stroke/MI Symptom Awareness and BP/Blood Glucose Awareness: proportion of adults who correctly identified ≥1 warning symptom or were aware of their own value, from Korea Disease Control and Prevention Agency (KDCA) Community Health Survey. Breast/Cervical Cancer Screening counts: number of individuals aged ≥55 years with suspected abnormal results from national cancer-screening programs (administrative screening outcomes, not confirmed diagnoses). Cancer screening data sourced from NHIS health-screening statistical yearbook via KOSIS. — : not available for the given year.
